# FIRE Study: Real-World Effectiveness and Safety of Ibrutinib in Clinical Practice in Patients with CLL and MCL

**DOI:** 10.1007/s44228-022-00015-5

**Published:** 2022-09-14

**Authors:** Caroline Dartigeas, Borhane Slama, Margaret Doyle, Christoph Tapprich, Claire Albrecht, Sandrine Dupuis, Robert Wapenaar, Charlotte Schmidt-Hieber, Veronique Leblond

**Affiliations:** 1 Service Hématologie et Thérapie Cellulaire, Hôpital Bretonneau, Tours, France; 2 CH Henri Duffaut, Avignon, France; 3 Janssen Sciences Ireland, Dublin, Ireland; 4 EMEA Medical Affairs, Janssen-Cilag, Neuss, Germany; 5 Janssen France, Issy-les-Moulineaux, France; 6 Janssen-Cilag B.V., Breda, The Netherlands; 7 APHP, Hôpital Saint-Louis, Hemato-Oncologie, Université de Paris, Paris, France; 8 Hematology Department, Hopital Pitie-Salpêtrière, Paris, France

**Keywords:** Ibrutinib, Chronic lymphocytic leukemia, Mantle cell lymphoma, Real-world evidence, Effectiveness, Safety

## Abstract

The FIRE study investigated the real-world effectiveness and safety of ibrutinib in prospectively observed patients with chronic lymphocytic leukemia (CLL)/small lymphocytic lymphoma (SLL) and mantle cell lymphoma (MCL) in France. Patients were mostly relapsed/refractory with high-risk features. First-line CLL/SLL patients had del17p and/or *TP53* mutations. In this interim analysis, the median follow-up time for patients with CLL/SLL and MCL was 17.7 and 15.1 months, respectively. In the effectiveness populations for CLL/SLL (*n* = 200) and MCL (*n* = 59), the median progression-free survival was not estimable and 12.4 months, respectively; the 12-month overall survival rates were 88.5% and 65.8%, respectively. Treatment-emergent adverse events of interest for patients with CLL/SLL (*n* = 202) and MCL (*n* = 59) included: infections and infestations (53.5% and 32.2%), major bleeding (5.0% and 5.1%), and atrial fibrillation (5.9% and 8.5%); 135 (66.8%) and 20 (33.9%) patients were continuing treatment at the time of data cutoff. Future analyses will report on longer-term follow-up (Trial registration: ClinicalTrials.gov, NCT03425591. Registered 1 February 2018—Retrospectively registered, https://clinicaltrials.gov/ct2/show/NCT03425591).

**Supplementary Information:**

The online version contains supplementary material available at 10.1007/s44228-022-00015-5.

## Introduction

Ibrutinib, a first-in-class, once-daily oral Bruton’s tyrosine kinase inhibitor (BTKi), is approved in Europe as monotherapy for the treatment of adults with chronic lymphocytic leukemia (CLL) and those with mantle cell lymphoma (MCL) who have received ≥ 1 prior line of therapy [1].

In phase 3 trials, single-agent ibrutinib has proven effective in treating patients with CLL, offering improved progression-free survival (PFS) and overall survival (OS) versus chlorambucil in previously untreated patients (RESONATE-2™, NCT01722487) [2] and improved PFS and OS versus ofatumumab in relapsed/refractory (R/R) patients (RESONATE™, NCT01578707) [3]. Several trials, including Alliance (ibrutinib alone or ibrutinib plus rituximab versus bendamustine plus rituximab, NCT01886872), HELIOS (ibrutinib plus bendamustine and rituximab, NCT01611090), and iLLUMINATE (ibrutinib plus obinutuzumab versus chlorambucil plus obinutuzumab, NCT02264574) demonstrated both PFS and OS benefit in patients with CLL [4–6]. Similarly, trials of ibrutinib as part of combination therapy, such as ECOG-1912 (previously untreated patients receiving ibrutinib plus rituximab versus combined fludarabine, cyclophosphamide, and rituximab [FCR], NCT02048813) showed that ibrutinib was the only BTKi to demonstrate benefit in PFS and OS versus FCR [7].

Likewise, in registered clinical trials in patients with R/R MCL (PCYC-1104, NCT01236391; RAY, NCT01646021) [8, 9], single-agent ibrutinib provided high response rates, demonstrated 2-year PFS of 31–41%, and was well tolerated. In a pooled analysis of three studies (SPARK, NCT01599949; RAY, NCT01646021; PCYC-1104, NCT01236391), ibrutinib appeared to mitigate the historical trend of successive declines in median PFS, seen with each successive line of chemoimmunotherapy, regardless of prior line of treatment and efficacy in first-line therapy [10]. Furthermore, ibrutinib therapy administered earlier in the treatment pathway significantly improved PFS [10].

While a large number of studies have shown the high efficacy of ibrutinib in a trial setting [2–9], the retrospective and prospective, noninterventional, multicenter FIRE study (NCT03425591) aimed to investigate the real-world effectiveness and safety of ibrutinib in patients with previously untreated and R/R CLL/SLL and R/R MCL in France, including those with high-risk features.

We report the results of the second interim analysis (data cutoff: August 30, 2018) of the prospectively included patients with CLL/SLL and MCL with 17.4- and 15.1-month follow-up, respectively.

## Materials and Methods

The study started on May 12, 2016, and ended in July 2022. Only the prospectively observed patients with CLL/SLL and MCL were included in this analysis. Patients who initiated ibrutinib ≤ 30 days before enrollment in the study were selected as prospectively followed patients. Patients were treated according to usual clinical practice, with follow-up planned for up to 5 years. This interim analysis was based on patients with CLL/SLL and MCL in the effectiveness population with at least 12 months of treatment follow-up (or who permanently discontinued ibrutinib within 12 months).

### Inclusion Criteria

Eligible patients were ≥ 18 years and treated with ibrutinib. A confirmed diagnosis of CLL/SLL was required in those previously untreated patients with a del17p and/or *TP53* mutation, or in those who received ≥ 1 prior line of therapy, or a confirmed diagnosis of MCL with R/R disease. Patients were included according to marketing authorization in France in 2016, when reimbursement for ibrutinib treatment in patients with R/R CLL or previously untreated CLL with del17p/*TP53* mutations became available in France.

### Exclusion Criteria

Patients were excluded if they participated in the French ibrutinib early access (Autorisation Temporaire d’Utilisation) program because this population is generally more heavily pretreated and not representative of the general population in routine clinical practice.

### Outcome Measures

The effectiveness population included patients who met all inclusion criteria and who received ≥ 1 dose of ibrutinib. The safety population included all patients who received ≥ 1 dose of ibrutinib. The primary effectiveness endpoint was investigator-assessed PFS. Secondary effectiveness endpoints included treatment response and OS. PFS was defined as the time from ibrutinib initiation to progression or death from any cause. OS was defined as the time from ibrutinib initiation to the date of death from any cause. Response assessments were analyzed as binary (two data levels) and time-to-event endpoints. The overall response rate (ORR) was defined as the proportion of patients with at least an objective response (i.e. complete response [CR] or partial response [PR], or PR with lymphocytosis for patients with CLL), as assessed by the participating physician. The safety analyses included treatment exposure and assessment of treatment-emergent adverse events (TEAEs), particularly bleeding, major bleeding (defined as a severe and/or serious bleeding event), serious TEAEs, and TEAEs of special interest. Data are descriptive and have been reported for the total prospective patient population.

## Results

### Patient Characteristics

Of 202 patients with CLL/SLL in the safety population, 200 were included prospectively in the effectiveness population (Table 1). The median age was 72 years; 69.5% of patients were male. Of the assessed patients (*n* = 163), 52.1% had an Eastern Cooperative Oncology Group (ECOG) performance score (PS) of ≥ 1. Medical history or comorbidities were reported in 47.5% of patients. All the previously untreated patients (17.5%) had a del17p and/or *TP53* mutation. Of patients who underwent cytogenetic assessment, 58.6% (78/133) had del17p and/or *TP53* mutation and 28.6% (38/133) had del11q. Of patients assessed for *IGHV* mutations (*n* = 29) and complex karyotype (defined as ≥ 3 mutations) (*n* = 100), 72.4% (21/29) and 62.0% (62/100) had unmutated *IGHV* and complex karyotype mutations, respectively. Of 13 patients in the effectiveness population with a cardiac history at inclusion, 8 (61.5%) received ≥ 1 anti-thrombotic treatment concomitantly with ibrutinib.

**Table 1:** Patient baseline characteristics^a^

Characteristics	CLL/SLL *n* = 200	R/R MCL *n* = 59
Age, median (range), years	72 (43–91)	73 (49–88)
Age, < 60	28 (14.0)	3 (5.1)
Age, 60–75	100 (50.0)	33 (55.9)
Age, > 75	72 (36.0)	23 (39.0)
Male	139 (69.5)	48 (81.4)
Time from diagnosis to ibrutinib initiation, median (range), years	7.2 (0.1–27.6)	4.0 (0.2–19.8)
Median time from stopping the prior line of therapy to treatment initiation of ibrutinib, median (range), months	26.0 (0.0–131.3)	8.3 (0.5–180.4)
Treatment-free period between last therapy and ibrutinib initiation, months	*n* = 149	*n* = 59
< 36	100 (67.1)	48 (81.4)
≥ 36	49 (32.9)	11 (18.6)
Refractory to purine analog	*n* = 162 11 (6.8)	N/A
Creatinine clearance < 30 mL/min	*n* = 191 3 (1.6)	*n* = 59 0
Creatinine clearance ≥ 30 mL/min and < 70 mL/min	*n* = 191 43 (22.5)	*n* = 59 15 (25.4)
Number of prior line of therapies		
0	35 (17.5)	0
1	71 (35.5)	27 (45.8)
2	55 (27.5)	20 (33.9)
≥ 3	39 (19.5)	12 (20.3)
Patients with prior stem cell transplant	N/A	15 (25.4)
ECOG PS	*n* = 163	*n* = 53
0	78 (47.9)	21 (39.6)
1	66 (40.5)	22 (41.5)
2	16 (9.8)	7 (13.2)
3	3 (1.8)	3 (5.7)
Comorbidities and past history	95 (47.5)	34 (57.6)
Ongoing malignancy	*n* = 33 7 (21.2)	*n* = 15 3 (20.0)
Ongoing active infection with hepatitis B or C	*n* = 5 1 (20.0)	*n* = 3 0 (0.0)
Ongoing autoimmune hemolytic anemia	*n* = 12 8 (66.7)	N/A
Ongoing atrial fibrillation	*n* = 22 6 (27.3)	*n* = 10 4 (40.0)
Other ongoing cardiovascular disease	*n* = 22 10 (45.5)	*n* = 10 7 (10.0)
Ongoing respiratory disease	*n* = 29 16 (55.2)	*n* = 6 4 (66.7)
Ongoing uncontrolled active systemic infection or grade 3–4 infection	*n* = 17 2 (11.8)	*n* = 2 0 (0.0)
Type of hematologic malignancy		
CLL	192 (96.0)	N/A
SLL	8 (4.0)	N/A
Ann Arbor stage at diagnosis	N/A	*n* = 48
Missing values, *n*	N/A	11
Stage I	N/A	3 (6.3)
Stage II	N/A	3 (6.3)
Stage III	N/A	3 (6.3)
Stage IV	N/A	39 (81.3)
sMIPI	N/A	*n* = 39
High	N/A	16 (41.0)
Intermediate	N/A	16 (41.0)
Low	N/A	7 (17.9)
del17p and/or mutated *TP53*	*n* = 133 78 (58.6)	*n* = 8 6 (75.0)
del11q present	*n* = 133 38 (28.6)	*n* = 11 2 (18.2)
del17p	*n* = 150 58 (38.7)	*n* = 11 4 (36.4)
*TP53* mutated	*n* = 121 51 (42.1)	*n* = 5 2 (40.0)
*IGHV* unmutated	*n* = 29 21 (72.4)	*n* = 2 1 (50.0)
Complex karyotype	*n* = 100 62 (62.0)	*n* = 14 10 (71.4)

All data presented as *n* (%) unless otherwise noted

*CLL* chronic lymphocytic leukemia; *ECOG PS* Eastern Cooperative Oncology Group performance status; *MCL* mantle cell lymphoma; *N/A* not applicable; *SLL* small lymphocytic lymphoma; *sMIPI* simplified prognostic index for advanced-stage mantle cell lymphoma

Of 59 patients with MCL in the safety population, all were included prospectively in the efficacy population (Table 1). Their median age was 73 years; 81.4% of patients were male. Almost half (45.8%) of the 59 patients had one prior line of therapy, 33.9% had two, and 20.3% had ≥ 3 prior lines of therapy. Of patients assessed (*n* = 53), 60.4% had an ECOG PS of ≥ 1 and 57.6% had a medical history or medical comorbidities. Of those patients assessed in our study (*n* = 39), the majority were classified with high- or intermediate-simplified prognostic index for advanced-stage mantle cell lymphoma (sMIPI). Of patients who underwent cytogenetic assessment (*n* = 8), 75% (6/8) had del17p and/or mutated *TP53*. Of patients who underwent assessment for del11q, *IGHV*, and complex karyotype mutations, 18.2% (2/11), 50.0% (1/2), and 71.4% (10/14) had del11q, unmutated *IGHV*, and complex karyotype mutations, respectively. Six of 8 (75%) patients in the interim analysis efficacy population with a cardiac history at inclusion had received ≥ 1 anti-thrombotic treatment concomitantly with ibrutinib.

### Effectiveness

**Fig. 1:**
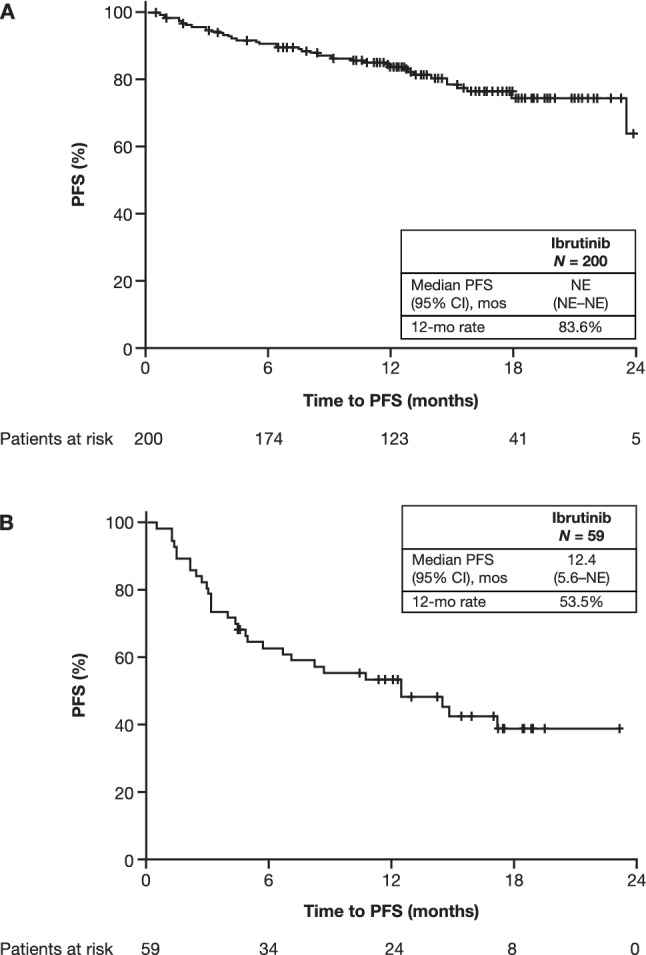
Impact of ibrutinib on PFS in patients with CLL/SLL (**A**) and patients with MCL (**B**). *CI* confidence interval; *CLL* chronic lymphocytic leukemia; *MCL* mantle cell lymphoma; *mo* month; *NE* not estimable; *PFS* progression-free survival; *SLL* small lymphocytic lymphoma

**Fig. 2:**
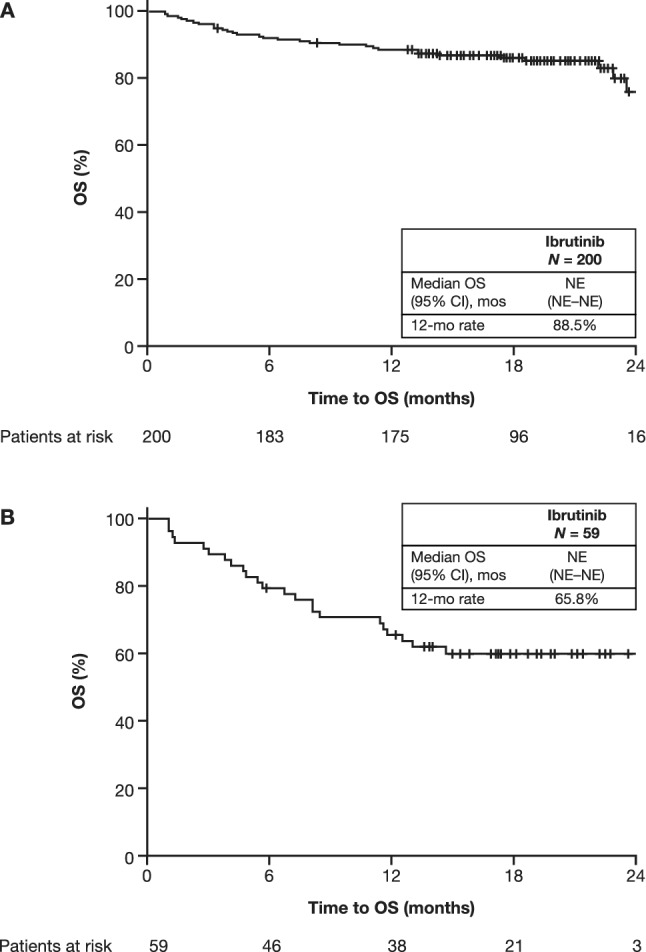
Impact of ibrutinib on OS in patients with CLL/SLL (**A**) and patients with MCL (**B**). *CI* confidence interval; *CLL* chronic lymphocytic leukemia; *MCL* mantle cell lymphoma; *mo* month, *NE* not estimable; *OS* overall survival; *SLL* small lymphocytic lymphoma

In the CLL/SLL patient cohort with a median follow-up of 17.7 (range, 0.1–27.2) months, ibrutinib treatment resulted in a median PFS and a median duration of response that were both not estimable (Fig. 1A; Table 2). The 12-month OS rate was 88.5% (Fig. 2A). The ORR was 94.5%, including a CR and PR with lymphocytosis reported in 22.1% and 72.4% of patients, respectively (Table 2). Subsequent therapy was initiated in 28 (13.9%) patients, with the most common (in ≥ 3 patients) being venetoclax (*n* = 11), ibrutinib (started again as next therapy after ibrutinib had been stopped for 3 months [*n* = 3]), rituximab plus cyclophosphamide, doxorubicin, vincristine, and prednisone (R-CHOP)/R-miniCHOP (*n* = 6), and rituximab plus idelalisib (*n* = 3).

**Table 2:** Survival and best response

	CLL/SLL	MCL
Survival	*n* = 200	*n* = 59
Median PFS (95% CI), months	NE (NE–NE)	12.4 (5.6–NE)
Median PFS for 0 lines of previous therapy (95% CI) months	23.5 (NE–NE)	N/A
Median PFS for 1 line of previous therapy (95% CI) months	24.2 (NE–NE)	17.1 (4.34–NE)
Median PFS for ≥ 2 lines of previous therapy (95% CI) months	NE (NE–NE)	8.4 (4.37–NE)
Median OS (95% CI), months	NE (NE–NE)	NE (NE–NE)
Best response, *n* (%)	*n* = 181	*n* = 51
Overall response	171 (94.5)	40 (78.4)
Complete response	40 (22.1)	21 (41.2)
Partial response (including partial response with lymphocytosis for CLL)	131 (72.4)	19 (37.3)
Stable disease	5 (2.8)	5 (9.8)
Progressive disease	5 (2.8)	6 (11.8)
Time to best response	*n* = 200	*n* = 59
Median (95% CI), months	3.5 (3.1–5.1)	3.9 (3.0–5.6)
Response duration	*n* = 200	*n* = 59
Median (95% CI), months	NE (NE–NE)	NE (NE–NE)

*CI* confidence interval; *CLL* chronic lymphocytic leukemia; *MCL* mantle cell lymphoma; *N/A* not applicable; *NE* not estimable; *OS* overall survival; *PFS* progression-free survival; *SLL* small lymphocytic lymphoma

In the R/R MCL patient cohort, with a median follow-up of 15.1 (range, 1.1–25.7) months, ibrutinib treatment resulted in a median PFS of 12.4 months and a median duration of response that was not estimable (Fig. 1B; Table 2). The 12-month OS rate was 65.8% (Fig. 2B). The ORR was 78.4%, with a CR and PR reported in 41.1% and 37.3% of patients, respectively (Table 2). A first subsequent therapy was initiated in 21 (35.6%) patients, with the most common (in ≥ 2 patients) being fludarabine (*n* = 2), R-CHOP/R-miniCHOP (*n* = 2), rituximab, cisplatin, dexamethasone, high-dose cytarabine (R-DHAP)/rituximab, dexamethasone, cytarabine, and oxaliplatin (R-DHAX)/rituximab, dexamethasone, high-dose cytarabine and carboplatin (DHAC) (*n* = 2 [all regimens]), and combined intravenous (IV) rituximab, IV bendamustine, subcutaneous velcade, IV dexamethasone (*n* = 2).

### Safety

In the CLL/SLL safety population, 185 of 202 (91.6%) patients initiated ibrutinib at the recommended daily dosage of 420 mg. The median (range) duration of therapy was 16.5 (0.1–27.2) months, 135 patients (66.8%) were still on treatment at this interim analysis, and 148 (73.3%) had no dose modifications. Of patients who temporarily interrupted ibrutinib (*n* = 65), the majority had one temporary interruption (*n* = 40 [61.5%]), which lasted for a median duration of 9 days. At the time of data cutoff, 135 (66.8%) patients remained on treatment, with patients permanently discontinuing ibrutinib due to toxicity (12.9%), disease progression (7.4%), death (4.0%), patient’s preference (0.5%), physician’s preference (0.5%), comorbidities (1.5%), or other reasons (4.0%). The most frequently occurring class of TEAEs was blood and lymphatic disorders (57.9% and 26.7% for overall and severe TEAEs, respectively). Severe TEAEs are listed in Supplementary Table 1 and TEAEs of interest are detailed in Table 3. Among the latter, infections and infestations were reported in 108 patients (53.5%; Supplementary Table 2). All-severity bleeding TEAEs were reported in 88 (43.6%) patients overall; 10 (5.0%) patients experienced ≥ 1 major bleeding TEAE. Among 31 patients who had ≥ 1 anti-thrombotic treatment, only one (3.2%) experienced major bleeding while on ibrutinib treatment. Atrial fibrillation (AF) and hypertension were reported in 12 (5.9%) and 21 (10.4%) patients, respectively.

**Table 3:** TEAEs of interest

*n* (%)	CLL/SLL *n* = 202	MCL *n* = 59
Patients with TEAEs (any severity)		
≥ 1 TEAE	197 (97.5)	59 (100)
≥ 1 TEAE related to ibrutinib	179 (88.6)	37 (62.7)
≥ 1 serious TEAE	102 (50.5)	41 (69.5)
≥ 1 severe TEAE	113 (55.9)	37 (62.7)
≥ 1 serious TEAE related to ibrutinib^a^	42 (20.8)	12 (20.3)
Patients with treatment-emergent bleeding events		
≥ 1 bleeding	88 (43.6)	20 (33.9)
≥ 1 major bleeding^b^	10 (5.0)	3 (5.1)
≥ 1 bleeding while on anti-thrombotic treatment^c^	21/31 (67.7)	11/20 (55.0)
≥ 1 major bleeding while on anti-thrombotic treatment^c^	1/31 (3.2)	1/20 (5.0)
Patients with ≥ 1 TEAE of interest (any severity)		
Infections and infestations^d^	108 (53.5)	19 (32.2)
Diarrhea	46 (22.8)	13 (22.0)
Arthralgia	30 (14.9)	7 (11.9)
Myalgia	19 (9.4)	2 (3.4)
Hypertension	21 (10.4)	3 (5.1)
Arrhythmia^d^	25 (12.4)	6 (10.2)
Atrial fibrillation	12 (5.9)	5 (8.5)
Atrial flutter	1 (0.5)	N/A
Rash^d^	8 (4.0)	4 (6.8)

*CLL* chronic lymphocytic leukemia; *MCL* mantle cell lymphoma; *N/A* not applicable; *SLL* small lymphocytic lymphoma; *TEAE* treatment-emergent adverse event

^a^Assessed by investigator

^b^Major bleeding is a severe/serious bleeding event

^c^Percentages are calculated based on the total number of patients with concomitant anti-thrombotic treatment

^d^Grouped terms

In the MCL safety population, at a median follow-up of 15.1 months, 48 of 59 (81.4%) patients initiated ibrutinib at the recommended dosage of 560 mg daily. The median (range) duration of therapy was 10.7 (0.4–25.7) months and 20 patients (33.9%) were still on treatment at the second interim analysis. Overall, 42 (71.2%) had no dose modifications. Of patients who temporarily interrupted ibrutinib (*n* = 20), the majority had one temporary interruption (15 [75.0%]), which lasted for a median duration of 10.5 days. At the time of data cutoff, 20 patients were continuing treatment, with patients permanently discontinuing ibrutinib due to disease progression (28.8%), death (13.6%), toxicity (8.5%), stem cell transplant (5.1%), or other reasons (8.5%). The most frequently occurring class of overall and severe TEAEs was general disorders and administration site conditions (69.5% and 25.4% for overall and severe TEAEs, respectively). Severe TEAEs are described in Supplementary Table 3 and TEAEs of interest are detailed in Table 3. Among the latter, infections and infestations were reported in 19 patients (32.2%; Supplementary Table 4). In total, 20 (33.9%) patients received ≥ 1 anti-thrombotic treatment. One third of patients (33.9%) reported ≥ 1 bleeding TEAE, of whom 11 (55.0%) were concomitantly treated with anti-thrombotic medication. Three (5.1%) patients reported ≥ 1 major bleeding TEAE, and of the 20 patients treated with concomitant anti-thrombotic medication, only 1 (5.0%) reported ≥ 1 major bleeding TEAE. AF and hypertension were reported in 5 (8.5%) and 3 (5.1%) patients, respectively.

## Discussion

FIRE is a real-world study evaluating the effectiveness and safety of ibrutinib in patients with CLL/SLL (who were predominantly R/R) and patients with R/R MCL who have high-risk features treated in routine clinical practice in France.

In our analysis, ibrutinib was initiated for CLL at the recommended dosage of 420 mg daily [1] for almost all patients (91.6%) with a median treatment duration of 16.5 months, a result of the short follow-up time. The 66.8% of patients with CLL who remained on treatment in our study was less than the 86% reported for single-agent ibrutinib in previously treated patients in the RESONATE clinical trial, a study with an R/R population comprising a similar percentage of patients with del17p mutations (32%) to those in our study (38.7%) [3]. However, a similar proportion of high-risk patients remaining on 420 mg daily single-agent ibrutinib treatment (57%) was reported in a previous phase 2 clinical study of previously untreated and R/R patients predominantly with *TP53* aberrations [11]. That same clinical study reported no dose modifications in 89.5% of high-risk patients, which is within the range reported in our study (73.3%) [11].

Ibrutinib was initiated for MCL at the recommended dosage of 560 mg daily [1] in the majority of the population (81.4%) with a median treatment duration of 10.7 months, again due to the short follow-up time. It is notable that 23.9% of patients with MCL remained on treatment in our study, which is substantially smaller than the percentage of R/R MCL patients with MCL in the phase 3 RAY trial who were treated with ibrutinib monotherapy at first relapse (46.8%).

The median PFS was not estimable in patients with CLL, as reported for single-agent ibrutinib in R/R patients in the RESONATE clinical trial [3]. Similarly, 6-year follow-up data from the same study continued to show this PFS benefit of ibrutinib (median PFS: 44.1 months) [12]. While the median OS in our study was not estimable, the high 12-month OS rate (88.5%) was supported by the rates reported in the RESONATE clinical study (90%), in the Danish multicenter, retrospective, real-world study cohort (88.8%) and in the UK Chronic Lymphocytic Leukaemia Forum real-world study (83.8%) [3, 13, 14]. The ORR determined in our study was somewhat higher than reported in RESONATE, but similar to the 6-month ORR reported in a phase 2 study with high-risk patients [3, 11].

The short PFS time in patients with MCL reported in our study (12.4 months) might have been influenced partly by the majority of patients who were assessed as having a high or intermediate sMIPI score and partly by the real-world analysis. The median PFS for patients with MCL was, nevertheless, similar to the PFS of 12.5 months reported for single-agent ibrutinib in MCL from the pooled analysis of three clinical studies (PCYC-1104 [a heavily pre-treated population], SPARK, phase 3 RAY) [15]. While the median OS in our study was not estimable, the high 12-month OS rate (65.8%) and ORR (78.4%) were similar to the corresponding rates reported in RAY (68% and 72%, respectively) [8].

The discontinuation rate due to toxicity observed in our study for patients with CLL (12.9%) is comparable to previous real-world reports: a Swedish retrospective study (20.0% [19/95]) [16], a Danish multicenter, retrospective cohort study (22.9% [47/205]) [13], the US-based CONNECT registry study (21.6% in patients with R/R CLL) [17], and a US-based single-center study (14.8%) [18], which are all higher than those reported in RESONATE (4%) [3].

In our study, the CLL population also reported low rates (5.0%) of major bleeding, comparable to those reported in RESONATE (1.0%) [3]. Furthermore, there was no increased risk of major bleeding when ibrutinib was given with anti-thrombotic therapy (3.2%). Rates of AF (5.9%) were broadly comparable to those reported in RESONATE (5.1%) [3]. Additionally, infections and infestations occurred in approximately half of the patients in our study (53.5%), which is notably less than the 70% reported in RESONATE.

These differences in the discontinuations due to TEAEs might relate to the TEAE management and treatment adherence strategies adopted in France [19, 20]. The results of a retrospective survey involving 11 French CLL treatment centers with a cohort of first-line and R/R patients receiving ibrutinib outside a clinical trial also noted higher rates of discontinuations versus clinical trials, possibly due to the lack of physicians’ experience with managing toxicity [21]. This same survey demonstrated that symptom monitoring significantly reduced ibrutinib discontinuation and improved OS, independent of age, line of treatment, and del17p/*TP53* mutations [21].

In patients with MCL, the discontinuation rate due to toxicity (8.5%) in our study is in the range reported in the phase 3 RAY trial (6.0%) and PCYC-1104 clinical trial (11%) [8, 9], and lower than the early discontinuations reported in a real-world Italian observational, retrospective study (16.9%) [22]. Discontinuation in MCL patients in our study was predominantly due to disease progression (28.8%). This finding is supported by the phase 3 RAY clinical study, which reported that disease progression was the main contributor to ibrutinib discontinuation (39.6% of the total population) [8]. In the MCL population, the major bleeding rate (5.1%) was comparable to the grade ≥ 3 major bleeding of 4.9% reported in the pooled analysis of three clinical studies [23], and was similar in the subset of patients who received ibrutinib concomitantly with anti-thrombotic therapy (5.0%). The AF rate (8.5%) was within the range reported in the phase 3 RAY (4.0%) and PCYC-1104 clinical trials (11%) [8, 9]. Infections and infestations occurred in 32.2% of patients, which is notably less than in the PCYC-1104 study (78%) [9] and MCL2002 study (87.5%) [24]. As with the patients with CLL/SLL in this study, the differences in the incidence of TEAEs and discontinuations due to TEAEs in patients with MCL might relate to the TEAE management and treatment adherence strategies adopted in France [19].

Of patients with CLL/SLL who underwent cytogenetic assessment in our study, 75%, 60.5%, and 66.5% received testing for del17p, mutated *TP53,* and del11q, respectively, yet only 15% of patients in the CLL/SLL population were tested for *IGHV*. Today, *IGHV* testing is more practice integrated for patients with CLL than at the start of the study in 2016; until recently, this test was only performed in clinical trials but is now becoming an important decision-making element in Europe. Lack of *IGHV* testing and/or incorrect interpretation can lead to patients not receiving appropriate treatment. In the observational informCLL registry of patients who received treatment for CLL/SLL, *IGHV* mutation status testing was performed in only 12% of patients; 71% of these had an unmutated gene, of which 39% received chemoimmunotherapy [25]*.* In addition, a Czech prospective observational study assessing safety and efficacy of low-dose FCR in elderly/comorbid patients showed that, while there was no difference in response between patients with mutated or unmutated *IGHV*, PFS was markedly longer in patients with mutated *IGHV* [26]. Testing for *IGHV* is advised in the recommendations of the French CLL Study Group and International Workshop on Chronic Lymphocytic Leukemia guidelines prior to first-line therapy [20, 27], which should help provide patients with optimal therapy moving forward. Complex karyotype was only assessed in 50% of patients in our study. While karyotyping is recommended, it is not mandatory for management in CLL [20]. While *IGHV* mutational status testing was performed in 10% of patients with MCL in our study, European guidelines state such testing is optional in patients with MCL [28].

In our study, rates of infection, bleeding, and AF were comparable to or lower than those reported in clinical trials [3, 8, 9, 23, 24]. In addition, no new safety signals were identified. The strengths of our study are, firstly, the inclusion of del17p/*TP53* in first-line CLL/SLL patients, a patient population not frequently studied in the real world. Secondly, our inclusion of elderly patients (86.0% and 94.0% ≥ 60 years in the CLL/SLL and MCL populations, respectively) and patients with renal impairment (24.1% and 25.4% of the assessed CLL/SLL and MCL populations) may be a more realistic representation of the actual treatment population, including many patients who would normally be excluded from clinical trials. Indeed, one retrospective analysis showed that only 32% of patients in phase 2 and 3 clinical trials were elderly, whereas 61% of cancer patients in the United States were elderly (≥ 65 years); this suggests that elderly patients may be underrepresented in cancer trials [29].

While interpreting our results, the following limitations should be considered. Our analysis is limited by the short median follow-up time. The number of patients in the CLL/SLL population who were tested for *IGHV* was very low versus current recommendations in France [20]. Unlike clinical trials, the effectiveness and safety parameters are presented through descriptive data in a real-world setting and assessed by the investigators; the response was assessed by physicians in routine clinical practice. Our real-world study will not have the same support from study teams and longer-term experience that impacts AE management as would be expected in a clinical trial. Finally, the dataset is too small to explore the effects of the line of therapy on PFS or the association of comorbidities on AF or bleeding risk. Nevertheless, these analyses may be feasible with longer follow-up data.

## Conclusion

In summary, ibrutinib treatment in this large real-world analysis was effective in patients who were mostly R/R with high-risk features, representing the clinical spectrum of a CLL population. Safety results are generally aligned with those reported from clinical trials [3, 8, 9, 23, 24] and other real-world studies in patients with CLL/SLL [13, 16–18] and MCL [22]. Furthermore, there was no increased risk of major bleeding when ibrutinib was given with anti-thrombotic therapy. Real-world data from FIRE are complementary to those from clinical trials and could potentially inform the sequence of treatment for specific risk groups within CLL/SLL and MCL populations. Additional follow-up is needed to confirm the effectiveness and safety of ibrutinib over the longer term.

## Supplementary Information

Below is the link to the electronic supplementary material.

Supplementary file1 (PDF 153 kb)

## Data Availability

The data-sharing policy of the Janssen Pharmaceutical Companies of Johnson & Johnson is available at www.janssen.com/clinical-trials/transparency. Requests for access to data from select studies can be submitted through the Yale Open Data Access (YODA) Project site at yoda.yale.edu.
