# Supplementary material for: FIRE Study: Real-World Effectiveness and Safety of Ibrutinib in Clinical Practice in Patients with CLL and MCL
Source: Clin Hematol Int. 2022 Sep 14;4(3):65–74. doi: 10.1007/s44228-022-00015-5 (PMC9492818; doi:10.1007/s44228-022-00015-5)
Supplement: Supplementary file 1 — Supplementary file1 (PDF 153 kb) [file 44228_2022_15_MOESM1_ESM.pdf]

Supplementary information for:

FIRE Study: Real-World Effectiveness and Safety of Ibrutinib in Clinical Practice in Patients With CLL and MCL

Clinical Hematology International

Caroline Dartigeas, Borhane Slama, Margaret Doyle, Christoph Tapprich, Claire Albrecht, Sandrine Dupuis, Robert Wapenaar, Charlotte Schmidt-Hieber, Veronique Leblond

Corresponding author: Caroline Dartigeas, Service Hématologie et Thérapie Cellulaire, Hôpital Bretonneau, Tours, France. Email: C.DARTIGEAS@chu-tours.fr

Supplementary Table 1 Most common (≥2% all events) severe TEAEs by system organ class and preferred term, split by relationship with ibrutinib: prospective CLL/SLL patients in safety population

System Organ Class<br>Preferred Term	Related	Not Related	All Events
**At least one severe TEAE**, *n* (%)	**72 (35.6)**	**64 (31.7)**	**110 (54.5)**
**Blood and lymphatic system disorders**, *n* (%)	**42 (20.8)**	**20 (9.9)**	**54 (26.7)**
Lymphocytosis	24 (11.9)	3 (1.5)	26 (12.9)
Thrombocytopenia	8 (4.0)	6 (3.0)	14 (6.9)
Neutropenia	9 (4.5)	7 (3.5)	13 (6.4)
Anemia	3 (1.5)	6 (3.0)	9 (4.5)
Hyperleukocytosis	7 (3.5)	1 (0.5%)	8 (4.0)
**Infections and infestations**, *n* (%)	**8 (4.0)**	**13 (6.4)**	**20 (9.9)**
Septic shock	3 (1.5)	3 (1.5)	6 (3.0)
**Investigations**, *n* (%)	**11 (5.4)**	**2 (1.0)**	**13 (6.4)**
Lymphocyte count increased	8 (4.0)	0	8 (4.0)
**Neoplasms benign, malignant and unspecified (including cysts and polyps)**, *n* (%)	**2 (1.0)**	**10 (5.0)**	**12 (5.9)**
Richter's syndrome	1 (0.5)	3 (1.5)	4 (2.0)
**Respiratory, thoracic, and mediastinal disorders**, *n* (%)	**3 (1.5)**	**9 (4.5)**	**12 (5.9)**
Lung disorder	2 (1.0)	6 (3.0)	8 (4.0)
**General disorders and administration site conditions**, *n* (%)	**5 (2.5)**	**5 (2.5)**	**10 (5.0)**
Asthenia	3 (1.5)	2 (1.0)	5 (2.5)
General physical health deterioration	1 (0.5)	3 (1.5)	4 (2.0)

**System Organ Class**<br>**Preferred Term**	**Related**	**Not Related**	**All Events**
**Cardiac disorders**, *n* (%)	**5 (2.5)**	**4 (2.0)**	**9 (4.5)**
Cardiac failure	3 (1.5)	1 (0.5)	4 (2.0)
**Nervous system disorders**, *n* (%)	**4 (2.0)**	**5 (2.5)**	**9 (4.5)**
**Musculoskeletal and connective tissue disorders**, *n* (%)	**2 (1.0)**	**6 (3.0)**	**8 (4.0)**
**Gastrointestinal disorders**, *n* (%)	**2 (1.0)**	**4 (2.0)**	**6 (3.0)**
**Injury, poisoning, and procedural complications**, *n* (%)	**1 (0.5)**	**4 (2.0)**	**5 (2.5)**
**Skin and subcutaneous tissue disorders**, *n* (%)	**3 (1.5)**	**2 (1.0)**	**5 (2.5)**
**Metabolism and nutrition disorders**, *n* (%)	**1 (0.5)**	**3 (1.5)**	**4 (2.0)**
**Surgical and medical procedures**, *n* (%)	0	**4 (2.0)**	**4 (2.0)**
**Vascular disorders**, *n* (%)	**2 (1.0)**	**2 (1.0)**	**4 (2.0)**

CLL, chronic lymphocytic leukemia; SLL, small lymphocytic lymphoma; TEAE, treatment-emergent adverse event.

Supplementary Table 2 Most common (≥1% all events) infections and infestation TEAEs in patients with CLL/SLL

**System Organ Class**<br>**Preferred Term**	**Related**	**Not Related**	**Total**
**Infections and infestations**, *n* (%)	**26 (12.9)**	**85 (42.1)**	**108 (53.5)**
Bronchitis, *n* (%)	3 (1.5)	23 (11.4)	26 (12.9)
Urinary tract infection, *n* (%)	0	11 (5.4)	11 (5.4)
Influenza, *n* (%)	0	7 (3.5)	7 (3.5)
Septic shock, *n* (%)	3 (1.5)	3 (1.5)	6 (3.0)
Sinusitis, *n* (%)	1 (0.5)	5 (2.5)	6 (3.0)
Erysipelas, *n* (%)	2 (1.0)	3 (1.5)	5 (2.5)
Fungal infection, *n* (%)	1 (0.5)	4 (2.0)	5 (2.5)
Rhinitis, *n* (%)	2 (1.0)	3 (1.5)	5 (2.5)
Ear infection, *n* (%)	0	4 (2.0)	4 (2.0)
Infection, *n* (%)	1 (0.5)	3 (1.5)	4 (2.0)
Nasopharyngitis, *n* (%)	0	4 (2.0)	4 (2.0)
Pharyngitis, *n* (%)	0	4 (2.0)	4 (2.0)
Sepsis, *n* (%)	1 (0.5)	3 (1.5)	4 (2.0)
Conjunctivitis, *n* (%)	0	2 (1.0)	3 (1.5)
Pneumonia, *n* (%)	0	2 (1.0)	3 (1.5)
Upper respiratory tract infection, *n* (%)	0	3 (1.5)	3 (1.5)
Cystitis, *n* (%)	0	2 (1.0)	2 (1.0)
Gastroenteritis, *n* (%)	0	2 (1.0)	2 (1.0)
Herpes zoster, *n* (%)	1 (0.5)	1 (0.5)	2 (1.0)

**System Organ Class**<br>**Preferred Term**	**Related**	**Not Related**	**Total**
Lung infection, *n* (%)	0	2 (1.0)	2 (1.0)
Onychomycosis, *n* (%)	1 (0.5)	1 (0.5)	2 (1.0)
Paronychia, *n* (%)	0	2 (1.0)	2 (1.0)
Pneumocystis jirovecii pneumonia, n (%)	2 (1.0)	0	2 (1.0)
Pneumonia pneumococcal, *n* (%)	1 (0.5)	1 (0.5)	2 (1.0)
Viral infection, *n* (%)	0	2 (1.0)	2 (1.0)

CLL, chronic lymphocytic leukemia; SLL, small lymphocytic lymphoma; TEAE, treatment-emergent adverse event.

Supplementary Table 3 Most common (≥2% all events) severe TEAEs by system organ class and preferred term split by relationship with ibrutinib: prospective MCL patients in safety population

System Organ Class<br>Preferred Term	Related	Not Related	All Events
**At least one severe TEAE**, *n* (%)	**12 (20.3)**	**30 (50.8)**	**36 (61.0)**
**General disorders and administration site conditions**, *n* (%)	**1 (1.7)**	**14 (23.7)**	**15 (25.4)**
General physical health deterioration	0	13 (22.0)	13 (22.0)
Asthenia	1 (1.7)	1 (1.7)	2 (3.4)
**Blood and lymphatic system disorders**, *n* (%)	**4 (6.8)**	**5 (8.5)**	**9 (15.3)**
Thrombocytopenia	1 (1.7)	3 (5.1)	4 (6.8)
Lymphocytosis	2 (3.4)	1 (1.7)	3 (5.1)
Neutropenia	2 (3.4)	1 (1.7)	3 (5.1)
Anemia	0	2 (3.4)	2 (3.4)
**Infections and infestations**, *n* (%)	**2 (3.4)**	**6 (10.2)**	**8 (13.6)**
**Respiratory, thoracic, and mediastinal disorders**, *n* (%)	**2 (3.4)**	**4 (6.8)**	**6 (10.2)**
Dyspnea	1 (1.7)	2 (3.4)	3 (5.1)
Lung disorder	0	2 (3.4)	2 (3.4)
**Cardiac disorders**, *n* (%)	**3 (5.1)**	**1 (1.7)**	**4 (6.8)**
Atrial fibrillation	2 (3.4)		2 (3.4)

**System Organ Class**<br>**Preferred Term**	**Related**	**Not Related**	**All Events**
**Injury, poisoning, and procedural complications**, *n* (%)	**1 (1.7)**	**2 (3.4)**	**3 (5.1)**
**Metabolism and nutrition disorders**, *n* (%)	0	**2 (3.4)**	**2 (3.4)**
Tumor lysis syndrome	0	2 (3.4)	2 (3.4)
**Nervous system disorders**, *n* (%)	0	**2 (3.4)**	**2 (3.4)**

MCL, mantle cell lymphoma; TEAE, treatment-emergent adverse event.

Supplementary Table 4 Infections and infestation TEAEs in patients with MCL

	All Events		
**System Organ Class**<br>**Preferred Term**	**Related**	**Not Related**	**Total**
**Infections and infestations**, *n* (%)	**5 (8.5)**	**17 (28.8)**	**19 (32.2)**
Bronchitis, *n* (%)	0	3 (5.1)	3 (5.1)
Urinary tract infection	1 (1.7)	3 (5.1)	3 (5.1)
Lung infection, *n* (%)	0	2 (3.4)	2 (3.4)
Pneumonia, *n* (%)	0	2 (3.4)	2 (3.4)
Bacterial sepsis, *n* (%)	0	1 (1.7)	1 (1.7)
Campylobacter gastroenteritis, *n* (%)	0	1 (1.7)	1 (1.7)
Conjunctivitis bacterial, *n* (%)	1 (1.7)	0	1 (1.7)
Enterobacter infection, *n* (%)	0	1 (1.7)	1 (1.7)
Escherichia sepsis, *n* (%)	0	1 (1.7)	1 (1.7)
Folliculitis, *n* (%)	0	1 (1.7)	1 (1.7)
Gastroenteritis, *n* (%)	0	1 (1.7)	1 (1.7)
Herpes zoster, *n* (%)	0	1 (1.7)	1 (1.7)
Hordeolum, *n* (%)	0	1 (1.7)	1 (1.7)
Infection, *n* (%)	0	1 (1.7)	1 (1.7)
Influenza, *n* (%)	0	1 (1.7)	1 (1.7)
Nocardiosis, *n* (%)	1 (1.7)	0	1 (1.7)
Paronychia, *n* (%)	1 (1.7)	1 (1.7)	1 (1.7)
Peritonitis, *n* (%)	0	1 (1.7)	1 (1.7)
Pneumocystis jirovecii pneumonia, *n* (%)	1 (1.7)	0	1 (1.7)

	All Events		
**System Organ Class**			
**Preferred Term**	**Related**	**Not Related**	**Total**
Sepsis, *n* (%)	0	1 (1.7)	1 (1.7)
Systemic candida, *n* (%)	0	1 (1.7)	1 (1.7)
Upper respiratory tract infection, *n* (%)	0	1 (1.7)	1 (1.7)

MCL, mantle cell lymphoma; TEAE, treatment-emergent adverse event.
